# Bioassay-Guided Isolation and Identification of Xanthine Oxidase Inhibitory Constituents from the Leaves of *Perilla frutescens*

**DOI:** 10.3390/molecules201017848

**Published:** 2015-09-25

**Authors:** Li-Na Huo, Wei Wang, Chun-Yu Zhang, Hai-Bo Shi, Yang Liu, Xiao-Hong Liu, Bing-Hua Guo, Dong-Mei Zhao, Hua Gao

**Affiliations:** 1 College of Pharmacy, Qingdao University, Qingdao 266021, Shandong, China; E-Mails: huolina0808@163.com (L.-N.H.); buckuper@163.com (Y.L.); liuxiaohong1043@163.com (X.-H.L.); guobinghua1214@126.com (B.-H.G.); 18764209579@163.com (D.-M.Z.); gaohuaqy@126.com (H.G.); 2 Health Education Institute of Changchun, Changchun 130021, Jilin, China; E-Mail: zcy20150821@sohu.com; 3 Institute of Changbai Mountain Resources, Jilin Academy of Chinese Medicine Sciences, Changchun 130012, Jilin, China; E-Mail: Shihaibo3901@163.com

**Keywords:** *Perilla frutescens* (L.) Britt., hyperuricemia, xanthine oxidase, polyphenols

## Abstract

Activity-directed fractionation and purification processes were employed to identify xanthine oxidase (XO) inhibitory compounds from the leaves of *Perilla frutescens*. The total extract was evaluated *in vitro* on XO inhibitory activity and *in vivo* in an experimental model with potassium oxonate-induced hyperuricemia in mice which was used to evaluate anti-hyperuricemic activity. The crude extract showed expressive urate-lowering activity results. Solvent partitioning of the total extract followed by macroporous resin column chromatography of the *n*-butanol extract yielded four extracts and eluted parts. Among them, only the 70% ethanol eluted part of the *n*-butanol extract showed strong activity and therefore was subjected to separation and purification using various chromatographic techniques. Five compounds showing potent activity were identified by comparing their spectral data with literature values to be caffeic acid, vinyl caffeate, rosmarinic acid, methyl rosmarinate, and apigenin. These results indicate that pending further study, these compounds could be used as novel natural product agents for the treatment of hyperuricemia.

## 1. Introduction

Hyperuricemia is characterized by an abnormally high level of uric acid in the blood due to a metabolic disorder on its production or on its excretion [1]. Increasing clinical reports have shown that hyperuricemia is associated with an increasing risk of not only gout, but also cardiovascular disorders, renal dysfunction, hyperlipidemia, hypertension, cancer, diabetes and metabolic syndromes [2,3,4,5]. Lifestyle modifications such as weight reduction, decreased dietary purine intake and alcohol consumption may help to decrease blood uric acid, but many patients will still need medication to control their hyperuricemia. Xanthine oxidase (XO) plays a key role in uric acid biosynthesis by converting hypoxanthine to xanthine and further converting xanthine to uric acid. Allopurinol, an XO inhibitor which is structurally related to xanthine, binds tightly to the active site of XO and thus causes XO inhibition. This drug has been used clinically for more than 40 years. Unfortunately, severe adverse effects in some patients, including fever, skin rashes, allergic reactions, hepatitis, and nephropathy limit the clinical use of allopurinol [6]. For this reason, xanthine oxidase inhibitors from natural products have been explored as viable, harmless, and nontoxic alternatives for the treatment of hyperuricemia [7,8,9].

*Perilla frutescens* (L.) Britt. (Lamiaceae), also known in Chinese as Zi Su, has been extensively used as a traditional medicine in Southeast Asian countries for centuries, especially in China, Japan, and Korea. In the 2010 edition of the Chinese Pharmacopeia [10], the dried leaf of *P*. *frutescens* is recorded as Perillae Folium, the dried stem of *P*. *frutescens* is recorded as Perillae Caulis, and the dried seed of *P*. *frutescens* is recorded as Perillae Fructus. Previous phytochemical studies on Perillae Folium have resulted in the isolation and identification of a variety of constituents classes, including monoterpenes, triterpenes, flavonoids, phenylpropanoids, and phenolic compounds [11,12,13]. In recent years, the leaves of this plant have drawn the attention of researchers and consumers due to their nutritional and health benefits such as hepatoprotective [14], hypolipidemic [15], antiallergic [16], anticancer [17], anti-inflammatory [18], and antioxidant activities [19]. Our preliminary screening study revealed that the aqueous extract of *P*. *frutescens* leaves had a potent XO inhibitory effect. Although it was reported that the ethanol extract of *P*. *frutescens* leaves possessed XO inhibitory capacity, and (*Z*,*E*)-2-(3,4-dihydroxyphenyl) ethenyl ester and (*Z*,*E*)-2-(3,5-dihydroxyphenyl) ethenyl ester were isolated as XO inhibitors [20], to our best knowledge, there are no reports in the literature revealing xanthine oxidase inhibitors of the aqueous extract of this plant. The aim of the present study was to investigate the broad spectrum active constituents through bioassay-guided fractionation techniques.

## 2. Results and Discussion

### 2.1. Hypouricemic Activities of Extract in Potassium Oxonate-Induced Hyperuricemic Mice

**Figure 1:**
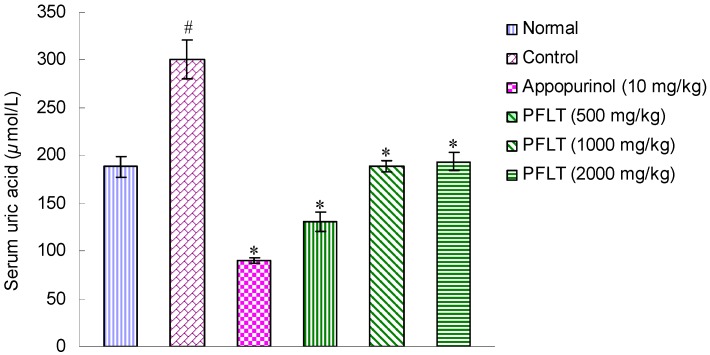
Effects of the water extracts of leaves from *P*. *frutescens* on serum urate levels in hyperuricemic mice pretreated with potassium oxonate. Control: Potassium oxonate alone. PFLT: An aqueous extract from the *P*. *frutescens* leaves. Data represent mean ± S.E.M. of 10 animals. For statistical significance, Student’s *t*-test was used between control and drug groups. ^#^ *p* < 0.05 when compared with normal group. * *p* < 0.05 when compared with control group.

Uricase inhibitor potassium oxonate treatment caused hyperuricemia in mice, as indicated by drastically increased serum uric acid levels [21]. The hyperuricemic effects of an aqueous extract from the *P*. *frutescens* leaves in potassium oxonate-induced hyperuricemic mice are shown in Figure 1. In normal mice, serum uric acid level was 188.1 ± 10.3 μmol/L. In hyperuricemic animals, serum uric acid levels were elevated to 300.7 ± 20.8 μmol/L 2 h after intraperitoneal injection of potassium oxonate. In contrast, the serum uric acid levels of the hyperuricemic group orally treated with the water extracts of *P*. *frutescens* leaves at doses of 500, 1000, and 2000 mg/kg for 7 days were significantly lowered to 193.7 ± 9.9, 188.7 ± 5.9, and 131.0 ± 10.4 μmol/L, respectively. Thus, the water extracts of *P*. *frutescens* leaves were able to induce an immediate decrease in serum uric levels in hyperuricemic mice.

### 2.2. Bioassay-Guided Isolation of Compounds 

**Figure 2:**
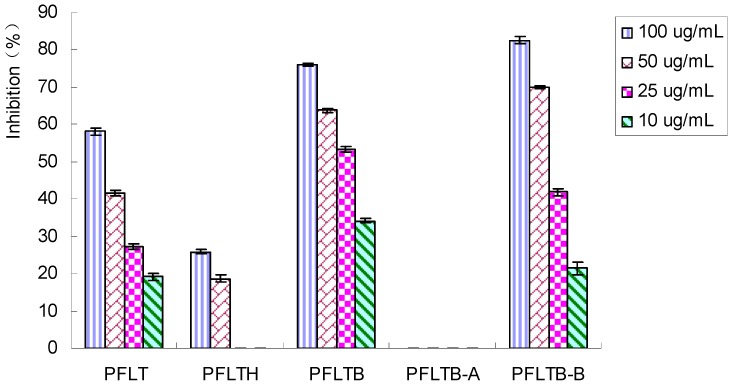
Xanthine oxidase inhibitory activities of the extracts and eluted parts by solvent and macroporous resin column separations. Data represent mean ± S.D. of triplicate experiments. PFLT: An aqueous extract from the *P*. *frutescens* leaves; PFLTH: Water extract; PFLTB: *n*-butanol extract; PFLTB-A: Water eluted part; PFLTB-B: 70% ethanol eluted part.

XO inhibitors from *P*. *frutescens* leaves were investigated using a bioassay-guided procedure to characterize active compounds for preventing and treating hyperuricemia. The enzyme activity has usually been measured spectrophotometrically by determing uric acid formation at 295 nm with xanthine as substrate [22,23]. Since direct spectrophotometry in UV region is prone to matrix interferences arising from the many organic compounds present in complex samples that also exhibit UV absorbance, uric acid formation was determined by an HPLC method in this study. Figure 2 summarises the XO inhibitory activity of different extracts *in vitro*, respectively.

**Figure 3:**
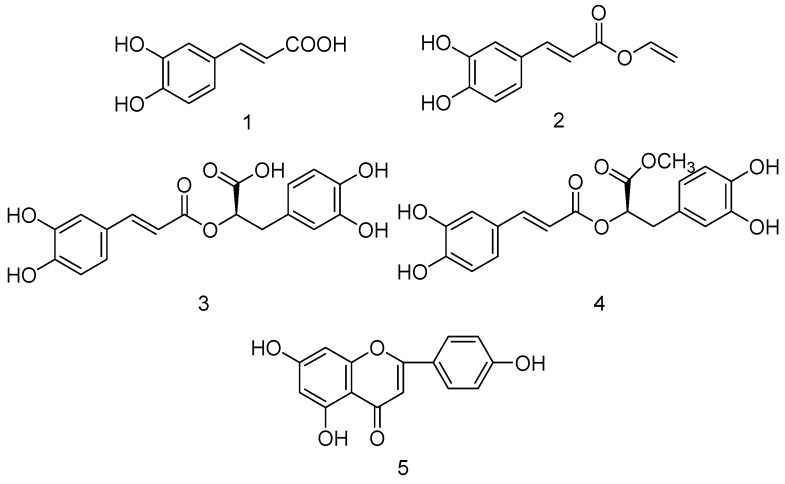
Structures of the compounds identified from active fractions: caffeic acid (**1**); vinyl caffeate (**2**); rosmarinic acid (**3**); methyl rosmarinate (**4**); apigenin (**5**).

The total extract was suspended in water and extracted with *n*-butanol giving *n*-butanol and water extracts. The inhibitory activity of the total extract was observed. The *n*-butanol extract which was obtained from the total extract also exhibited strong inhibitory activity against XO, while the water extract showed weaker inhibitory activity on XO. The *n*-butanol extract was subjected to HPD 600 macroporous resin column chromatography and eluted with water and 70% ethanol to give a water eluted part and 70% ethanol eluted part. From the results shown in Figure 2, the water eluted part did not show the desired inhibitory activity, while the 70% ethanol eluted part showed potential inhibitory activity. Hence, the 70% ethanol eluted part was subjected to activity-guided fractionation on an RP-18 reversed-phase silica gel column. After separation of active fractions by preparative HPLC, five compounds were obtained, and their structure were identified as caffeic acid (**1**) [24], vinyl caffeate (**2**) [24], rosmarinic acid (**3**) [25], methyl rosmarinate (**4**) [25], and apigenin (**5**) [26] on the basis of spectra data using ^1^H- and ^13^C-NMR and ESI-MS. The structures of the five compounds are shown in Figure 3.

### 2.3. Effect of Five Compounds on XO Activity and Inhibitory Modes of Action in Vitro 

#### 2.3.1. Effect on XO Activity

The assays were conducted to investigate whether various concentrations of the compounds isolated from active fractions inhibited the catalytic activity of XO. Allopurinol was used as positive control. Results showed that the IC_50_ values of the compounds **2**, **4**, and **5** exhibiting strong XO inhibitory activities *in vitro* were 31.26, 26.59, and 0.44 μM, respectively (Table 1). Compounds **1** and **3** exhibited low inhibitions against the activities of XO with IC_50_ values of 121.22 and 91.72 μM, respectively. Apigenin (compound **5**) with the minimum IC_50_ value inhibited XO in a concentration-dependent manner among the five compounds. In addition, the IC_50_ value of allopurinol, a clinical XO inhibitory drug, was 2.07 μM under the assay conditions. Among the four phenylpropanoid derivatives, the vinylation or methylation of carboxyl group of caffeic acid or rosmarinic acid appears to promote the XO inhibitory activity. Further detailed work is needed to clarify structure-activity relationship between different the esterified groups and XO inhibition.

**Table 1:** Xanthine oxidase inhibitory activity of the compounds isolated from active fractions.

Compound	IC50 (μM)	Ki (μM)	Mode of Inhibition
Caffeic acid	121.22	9.22	Competitive
Vinyl caffeate	31.26	5.05	Competitive
Rosmarinic acid	91.72	26.22	Competitive
Methyl rosmarinate	26.59	2.85	Competitive
Apigenin	0.44	0.11	Mixed
Allopurinol	2.07	2.42	Competitive

#### 2.3.2. Inhibition Kinetic Analysis

**Figure 4:**
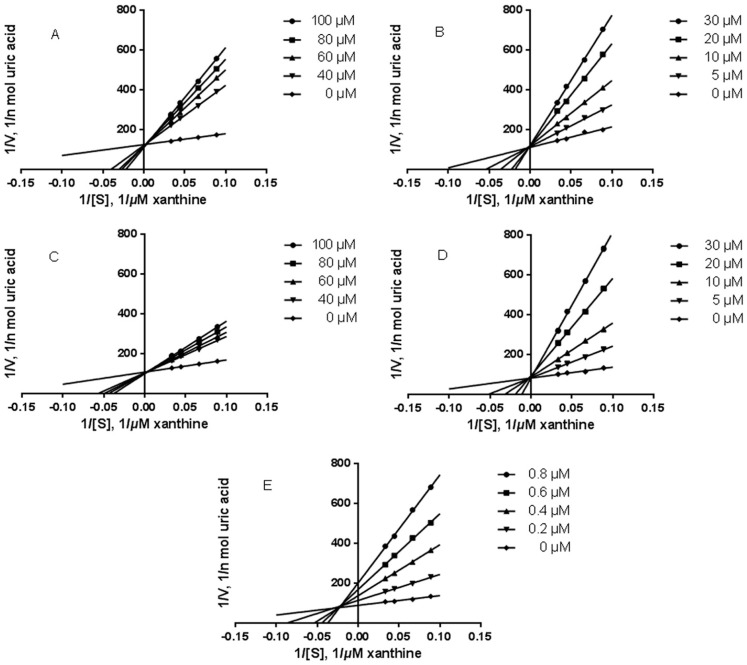
Lineweaver-Burk plots of XO inhibitions of compounds with various concentrations of xanthine. Lineweaver-Burk transformed data were plotted and followed by linear regression of the points. Data represent mean of triplicate experiments. (**A**) caffeic acid; (**B**) vinyl caffeate; (**C**) rosmarinic acid; (**D**) methyl rosmarinate; (**E**) apigenin.

Enzyme inhibition kinetic experiments were carried out to further characterize the inhibitory activities of the compounds isolated from active fractions. The inhibition modes of the compounds given in Table 1 were determined by analyzing the data by Lineweaver-Burk plots. For apigenin, a mixed inhibition mode was observed. The result suggested that apigenin inhibited XO activity not only by a competitive mode of action, but also by interaction with the enzyme at a site other than the active center which was accorded with the consequences in previous studies [27]. All other compounds and allopurinol exhibited competitive inhibitions. The Lineweaver-Burk plots in the absence or presence of compounds are shown in Figure 4. However, different types of inhibition by caffeic acid and its derivatives have been reported in the previous studies [20,27,28,29]. Under the experimental conditions, caffeic acid exhibited a competitive inhibition mode, which is consistent with the result reported by Chen *et al.* [28].

### 2.4. HPLC Analysis

**Figure 5:**
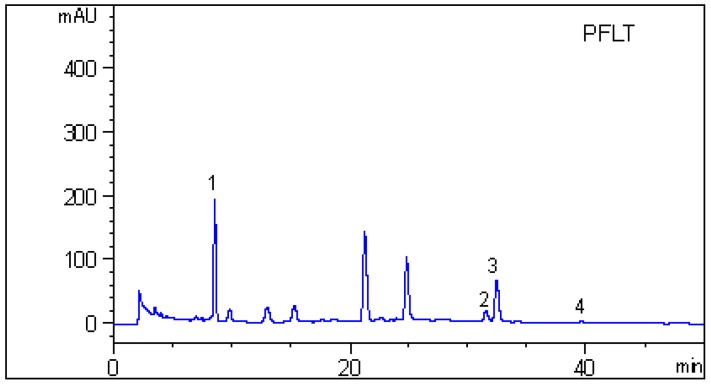

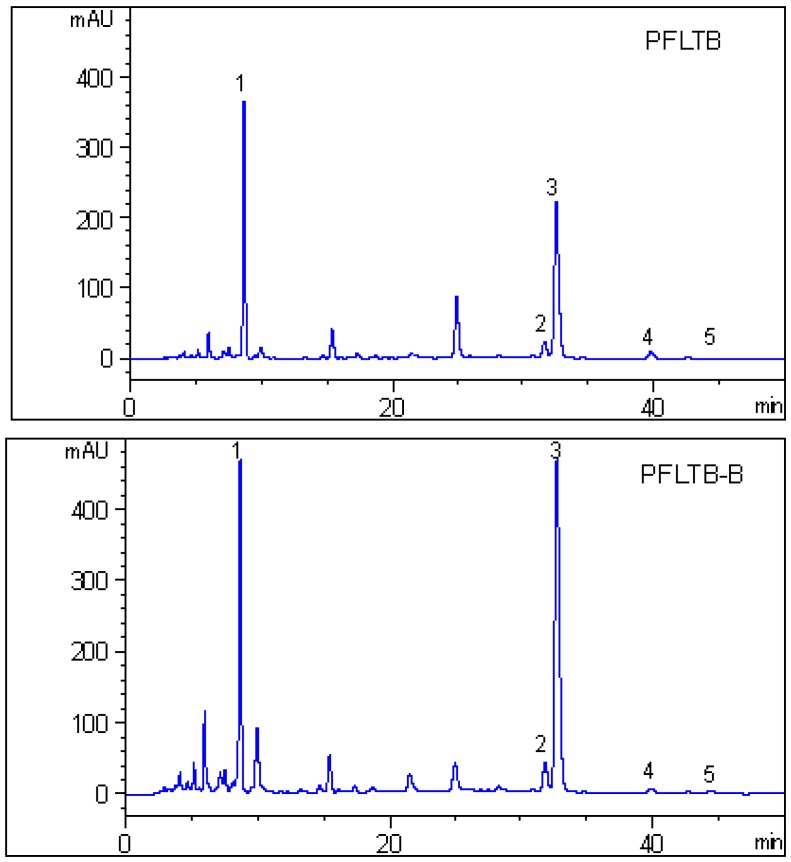
HPLC profiles of the active extracts and eluted part. PFLT: an aqueous extract from the *P*. *frutescens* leaves; PFLTB: *n*-butanol extract; PFLTB-B: 70% ethanol eluted part: caffeic acid (**1**); vinyl caffeate (**2**); rosmarinic acid (**3**); methyl rosmarinate (**4**); apigenin (**5**) were detected at 330 nm.

The HPLC profiles of active extracts and eluted part are shown in Figure 5. Though vinyl caffeate, methyl rosmarinate, and apigenin appeared as small peaks in the HPLC chromatogram of the 70% ethanol eluted part, the xanthine oxidase inhibitory activity assay showed that those compounds could significantly inhibit the production of uric acid, and the caffeic acid and rosmarinic acid responsible for major peaks exhibited weak XO inhibitory activities. However, in respect of its high content in the 70% ethanol eluted part, caffeic acid together with rosmarinic acid are likely candidates to contribute significantly to the XO inhibitory effect.

## 3. Experimental Section

### 3.1. General Procedures

ESI-MS was obtained with a Bruker micro-TOFQ mass spectrometer (Bruker, Bremen, Germany). NMR spectra were acquired on a Bruker AV-500 FT-NMR, and the chemical shifts were referenced to the residual solvent signals. Column chromatography was performed with macroporous resin HPD 600 (Cangzhou Bon Adsorber Technology Co., Ltd, Cangzhou, China) and RP-18 reversed-phase silica gel (S-50 mm; YMC, Kyoto, Japan). Preparative HPLC was performed using a Shimadzu LC-6AD pump connected to a Shimadzu SPD-20A UV-Vis detector (at 254 nm) with a Shim Pak ODS column (250 mm × 21.2 mm, i.d., 5 μm, Shimadzu, Kyoto, Japan). TLC was conducted on pre-coated TLC plates with silica gel RP-18 60 F254 (0.25 mm, Merck, Darmstadt, Germany). Detection was achieved by spraying the sample with 10% H_2_SO_4_ in MeOH followed by heating. HPLC grade MeOH was purchased from Merck. HPLC-grade water was purified using a Milli-Q system (Millipore, Boston, MA, USA). All solvents used for the chromatographic separations were distilled before use. Allopurinol, potassium oxonate, XO, and xanthine were bought from Sigma-Aldrich Chemicals (St. Louis, MO, USA). Assay kit of uric acid was obtained from Jiancheng Biotech (Nanjing, China).

### 3.2. Plant Materials

The raw materials of the leaves of *Perilla frutescens* were collected from Changchun suburb, Jilin Province of China, in October 2011, and authenticated by Bao-Min Feng, College of Life Science and Technology, Dalian University. A voucher specimen (ZS20111001) was deposited at the Institute of Phytochemistry, Jilin Academy of Chinese Medicine Sciences.

### 3.3. Animals 

The animal protocols used in this work were evaluated and approved by the Animal Use and Ethic Committee of Jilin Academy of Chinese Medicine Sciences (2012-A-0326). All experimental procedures were conducted in accordance with Guidelines for the Care and Use of Laboratory Animals. Male ICR mice (26–30 g) were obtained from Changchun Gaoxin Medical Animal Experimental Research Center, kept in cages, and exposed to a 12 h light/dark cycle. The room temperature and humidity were maintained automatically at approximately 25 °C and 60%, respectively. They were allowed at least 1 week to adapt to their environment before used for experiments and given a standard chow and water *ad libitum* for the duration of the study.

### 3.4. Extraction and Isolation of Bioactive Compounds

*P*. *frutescens* leaves (3 kg) were cut into small pieces and extracted with boiling water (36 L × 2 times) for 2 h and then filtered through a two-layer mesh. The water filtrates were collected and concentrated on a boiling bath to dryness producing 472 g of extract (PFLT). Part of the PFLT was reserved for activity assays, whilst the rest (400 g) was suspended in water and partitioned with *n*-butanol, followed by concentration to yield 88 g of *n*-butanol extract (PFLTB) and 298 g of water extract (PFLTH), respectively. The XO inhibitory activities of all the extracts were estimated by the *in vitro* method and the PFLTB extract showed strong activity when compared with the PFLT and PFTLH extract. The active PFLTB extract (65 g) was subjected to open column chromatography on HPD 600 macroporous resin and successively eluted with water and 70% ethanol, thus affording a water eluted part (PFLTB-A, 11 g) and a 70% ethanol eluted part (PFLTB-B, 49 g).

On the basis of the XO inhibitory activity, the part eluted with 70% ethanol was chromatographed over an RP-18 reversed-phase silica gel column (3 × 25, 100 g), eluting with a gradient of increasing MeOH (20%–100%) in water and separated into seven fractions (Fractions 1–7) on the basis of TLC analyses. Fractions 2, 6 and 7 exhibited significantly XO inhibitory activity. Fraction 2 was isolated by preparative HPLC (ODS column: 250 mm × 21.2 mm, i.d., 5 μm; flow rate: 2.0 mL/min) using MeOH–H_2_O (20:80) as the mobile phase to yield compound **1** (86.5 mg). Compounds **2** (9.2 mg) and **3** (52.8 mg) were obtained from fraction 6 by preparative HPLC (RP-18 column: 250 mm × 21.2 mm, i.d., 5 μm; flow rate: 2.0 mL/min) employing MeOH–H_2_O (48:52) as the mobile phase. Fraction 7 was purified by preparative HPLC (ODS column: 250 mm × 21.2 mm, i.d., 5 μm; flow rate: 2.8 mL/min) using MeOH–H_2_O (55:45) as the mobile phase to yield compounds **4** (4.7 mg) and **5** (3.9 mg).

### 3.5. Mice Model of Hyperuricemia and Drug Administration

All the mice were divided at random into six groups of ten mice each. The experimental animal model of hyperuricemia induced by uricase inhibitor potassium oxonate has been used to study drug action [28]. Briefly, the aqueous extract from the *P*. *frutescens* leaves (2000, 1000, and 500 mg/kg) and allopurinol (10 mg/kg) were dissolved in 0.3% CMC-Na aqueous solution, respectively. All drugs were orally administered once daily at 8:00–9:00 a.m. for seven consecutive days. Food, but not water, was withdrawn from the animals 1.5 h prior to drug administration, mice were intraperitoneally injected with potassium oxonate (280 mg/kg) 1h before the final drug administration to increase the serum urate level. Blood samples were collected from mice by tail vein bleeding 1 h after the final drug administration on the seventh day. The blood was allowed to clot for approximately 1 h at room temperature and then centrifuged at 3000× *g* for 10 min to obtain the serum. The serum was stored at −20 °C until use. Serum uric acid was measured using standard diagnostic kits. Each assay was performed in triplicate.

### 3.6. Assay of XO Activity and Inhibitory Mode of Action in Vitro 

#### 3.6.1. Assay of XO Activity

The XO inhibition assay was performed according to the method modified by our group [30]. The tested samples were dissolved in dimethyl sulfoxide (DMSO) and subsequently diluted with 70 mM phosphate buffer (pH = 7.5) to a final concentration containing less than 1% DMSO (*v*/*v*). The assay mixture consists of 200 μL of test solution, 140 μL of 70 mM phosphate buffer (pH = 7.5), and 120 μL enzyme solution (0.02 units/mL in the same buffer), which was prepared immediately before use. After preincubation at 25 °C for 15 min, the reaction was initiated by the addition of 240 μL of substrate solution (300 μM xanthine in the same buffer). The assay mixture was incubated at 25 °C for 30 min. The reaction was stopped by adding 100 μL of 1 M HCl, and uric acid level was determined by an HPLC method using a Diamonsil ODS-C18 column (250 × 4.6 mm i.d., 5 μm, Dikma Technologies, Beijing, China) on an Agilent 1260 system equipped with a G1311C quaternary pump, a G1329B autosampler, a G1316A thermostatted column compartment, and a G1314F variable wavelength detector coupled with an analytical workstation (Agilent Technologies, Inc., Santa Clara, CA, USA). After filtration, 20 μL of the sample was injected into the column and eluted with 100 mM sodium dihydrogen phosphate in water at a flow rate of 1 mL/min. The eluate was monitored for absorbance at 290 nm. Allopurinol was used as a positive control. The IC_50_ values of the samples were calculated from regression lines of a plot of the percentage of inhibition on XO activity *vs.* the concentrations of the samples.

#### 3.6.2. Assay of XO Inhibitory Modes of Action

Enzyme kinetics were determined in the absence and presence of the tested samples with varying concentrations of xanthine (37.5, 50, 75, and 100 μM) as the substrate, where tested samples were at various concentration (5–100 μM) and xanthine at a certain concentration, using the XO assay methodology. The plots were drawn by the reciprocal velocity (ν-1) *vs.* the concentration of the substrate (1/[xanthine]).

### 3.7. HPLC Analysis

The HPLC analyses were performed using the Agilent 1260 system mentioned above. The chromatographic separation was carried out on a ZORBAX Eclipse XDB (250 mm × 4.6 mm, 5 µm, Agilent Technologies, Inc.). The mobile phase consisted of methanol (A) and water containing 0.1% phosphoric acid (B). A gradient program was used: 0–10 min, isocratic elution with A–B (30:70, *v*/*v*); 10–25 min, linear change from A–B (30:70, *v*/*v*) to A–B (40:60, *v*/*v*); and 25–50 min, isocratic elution with A–B (40:60, *v*/*v*). The flow rate was 1.0 mL/min, and the column temperature was maintained at 25 °C. The product was detected by monitoring UV absorption at 330 nm. The samples were filtered through a 0.45 µm nylon membrane filter, and 20 µL were injected for HPLC analysis.

### 3.8. Statistical Analysis

Data from the *in vivo* experiments were expressed as the mean ± standard error of the mean (S.E.M.) and were analyzed by one-way analysis of variance using the SPSS version 13.0 software. A value of *p* < 0.05 was considered statistically significant. IC_50_ values of the samples were calculated with Origin version 8.0 software (OriginLab Corporation, Noethampton, UK). The inhibitory type and *K*_i_ value were analyzed using GraphPad Prism 5.0 software (GraphPad Software, Inc., La Jolla, CA, USA).

## 4. Conclusions

In summary, the results demonstrate for the first time that the anti-hyperuricemic activity of water extracts from *P*. *frutescens* leaves attributed to the reduction of uric acid production by inhibiting XO activity. Moreover, five compounds isolated from the active fractions and showing different XO inhibitory activities *in vitro* may contribute to the observed anti-hyperuricemic effect. These findings provide evidence of anti-hyperuricemic activity of *P*. *frutescens* leaves as a candidate health dietary source.

## References

[1] Punzi L., So A. (2013). Serum uric acid and gout: From the past to molecular biology. Curr. Med. Res. Opin..

[2] Puddu P., Puddu G.M., Cravero E., Vizioli L., Muscari A. (2012). The relationship among hyperuricemia, endothelial dysfunction, and cardiovascular diseases: Molecular mechanisms and clinical implications. J. Cardiol..

[3] Zoccali C., Mallamaci F. (2013). Uric acid, hypertension, and cardiovascular and renal complications. Curr. Hypertens. Rep..

[4] Choi H.K., Ford E.S. (2007). Prevalence of the metabolic syndrome in individuals with hyperuricemia. Am. J. Med..

[5] Behradmanesh S., Horestani M.K., Baradaran A., Nasri H. (2013). Association of serum uric acid with proteinuria in type 2 diabetic patients. J. Res. Med. Sci..

[6] Doghramji P.P. (2011). Managing your patient with gout: A review of treatment options. Postgrad. Med. J..

[7] Yu Z.F., Fong W.P., Cheng C.H. (2006). The dual actions of morin (3,5,7,2′,4′-pentahydroxyflavone) as a hypouricemic agent: Uricosuric effect and xanthine oxidase inhibitory activity. J. Pharmacol. Exp. Ther..

[8] Wang Y.J., Zhang G.W., Pan J.H., Gong D.M. (2015). Novel insights into the inhibitory mechanism of kaempferol on xanthine oxidase. J. Agric. Food Chem..

[9] Yan J.K., Zhang G.W., Hu Y.T., Ma Y.D. (2013). Effect of luteolin on xanthine oxidase: inhibition kinetic and interaction mechanism merging with docking simulation. Food Chem..

[10] State Pharmacopoeia Committee (2010). Pharmacopoeia of the People’s Republic of China.

[11] Woo K.W., Han J.Y., Suh W.S., Lee J.H., Lee K.R. (2014). Two new chemical constituents from leaves of *Perilla frutescens* var. acuta. Bull. Korean Chem. Soc..

[12] Banno N., Akihisa T., Tokuda H., Yasukawa K., Higashihara H., Ukiya M., Watanabe K., Kimura Y., Hasegawa J., Nishino H. (2004). Triterpene acids from the leaves of *Perilla frutescens* and their anti-inflammatory and antitumor-promoting effects. Biosci. Biotechnol. Biochem..

[13] Zhou X.J., Yan L.L., Yin P.P., Shi L.L., Zhang J.H., Liu Y.J., Ma C. (2014). Structural characterisation and antioxidant activity evaluation of phenolic compounds from cold-pressed *Perilla frutescens* var. arguta seed flour. Food Chem..

[14] Yang S.Y., Hong C.O., Lee H., Park S.Y., Park B.G., Lee K.W. (2012). Protective effect of extracts of *Perilla frutescens* treated with sucrose on tert-butyl hydroperoxide-induced oxidative hepatotoxicity *in vitro* and *in vivo*. Food Chem..

[15] Feng L.J., Yu C.H., Ying K.J., Hua J.A., Dai X.Y. (2011). Hypolipidemic and antioxidant effects of total flavonoids of *Perilla Frutescens* leaves in hyperlipidemia rats induced by high-fat diet. Food Res. Int..

[16] Oh H.A., Park C.S., Ahn H.J., Park Y.S., Kim H.M. (2011). Effect of *Perilla frutescens* var. acuta Kudo and rosmarinic acid on allergic inflammatory reactions. Exp. Biol. Med..

[17] Kwak Y., Ju J. (2015). Inhibitory activities of *Perilla frutescens* britton leaf extract against the growth, migration, and adhesion of human cancer cells. Nutr. Res. Pract..

[18] Urushima H., Nishimura J., Mizushima T., Hayashi N., Maeda K., Ito T. (2014). *Perilla frutescens* extract ameliorates DSS-induced colitis by suppressing proinflammatory cytokines and inducing anti-inflammatory cytokines. Am. J. Physiol. Gastrointest. Liver Physiol..

[19] Jun H.I., Kim B.T., Song G.S., Kim Y.S. (2014). Structural characterization of phenolic antioxidants from purple perilla (*Perilla frutescens* var. acuta) leaves. Food Chem..

[20] Nakanishi T., Nishi M., Inada H., Obata H., Abe S., Wakashiro M. (1990). Two new potent inhibitors of xanthine oxidase from leaves of *Perilla frutescens* Britton var. *acuta* kudo. Chem. Pharm. Bull..

[21] Stavric B., Clayman S., Gradd R.E., Hebert D. (1975). Some *in vivo* effects in the rat induced by chlorprothixene and potassium oxonate. Pharmacol. Res. Commun..

[22] Liu H.X., He M.T., Tan H.B., Gu W., Yang S.X., Wang Y.H., Li L., Long C.L. (2015). Xanthine oxidase inhibitors isolated from *Piper nudibaccatum*. Phytochem. Lett..

[23] Kim D.W., Curtis-Long M.J., Yuk H.J., Wang Y., Song Y.H., Jeong S.H., Park K.H. (2014). Quantitative analysis of phenolic metabolites from different parts of *Angelica keiskei* by HPLC–ESI MS/MS and their xanthine oxidase inhibition. Food Chem..

[24] Yao S., Xu N.Y., Chu C.J., Zhang J., Chen D.F. (2013). Chemical constituents of *Rabdosia japonica* var. glaucocalyx and their anti-complementary activity. Chin. J. Chin. Mater. Med..

[25] Sun Z.C., Zheng Q.X., Wu H.F., Ma G.X., Xu X.D., Yang J.S. (2014). Water-soluble constituents of *Clerodendranthus spicatus*. Chin. Pharm. J..

[26] Lee J.H., Park K.H., Lee M.H., Kim H.T., Seo W.D., Kim J.Y., Baek I.Y., Jang D.S., Ha T.J. (2013). Identification, characterisation, and quantification of phenolic compounds in the antioxidant activity-containing fraction from the seed of Korean perilla (*Perilla frutescens*) cultivars. Food Chem..

[27] Flemmig J., Kuchta K., Arnhold J., Rauwald H.W. (2011). *Olea europaea* leaf (Ph. Eur.) extract as well as several of its isolated phenolics inhibit the gout-related enzyme xanthine oxidase. Phytomedicine.

[28] Chen L.Y., Yin H.F., Lan Z., Ma S.W., Zhang C.F., Yang C.L., Li P., Lin B.Q. (2011). Anti-hyperuricemic and nephroprotective effects of *Smilax china* L.. J. Ethnopharmacol..

[29] Chiang H.C., Lo Y.J., Lu F.J. (1994). Xanthine-oxidase inhibitors from the leaves of *Alsophila spinulosa* (hook) *tryon*. J. Enzym. Inhib..

[30] Li X.C., Liu X.H., Gao H., Fan M.L., Liu K., Wang W. (2015). Study on inhibition and enzyme kinetics of different solvent extractions from *Polygonum cuspidatum* on xanthine oxidase. China Pharm..

